# Implications for medication safety and adherence in dermato‐oncology: The AMBORA care program for oral antitumor therapeutics

**DOI:** 10.1111/ddg.15809

**Published:** 2025-07-17

**Authors:** Lisa Cuba, Frank Dörje, Rafaela Kramer, Pauline Dürr, Michael Erdmann, Martin F. Fromm, Carola Berking, Katja Gessner

**Affiliations:** ^1^ Pharmacy Department Universitätsklinikum Erlangen Friedrich‐Alexander‐Universität Erlangen‐Nürnberg Erlangen Germany; ^2^ Institute of Experimental and Clinical Pharmacology and Toxicology Friedrich‐Alexander‐Universität Erlangen‐Nürnberg Erlangen Germany; ^3^ Comprehensive Cancer Center (CCC) Erlangen‐EMN Universitätsklinikum Erlangen Friedrich‐Alexander‐Universität Erlangen‐Nürnberg Erlangen Germany; ^4^ Bavarian Center for Cancer Research (BZKF) Erlangen Germany; ^5^ Pharmacy Department Clinic Floridsdorf Vienna Healthcare Group Vienna Austria (present address); ^6^ FAU NeW ‐ Research Center New Bioactive Compounds Friedrich‐Alexander‐Universität Erlangen‐Nürnberg Erlangen Germany; ^7^ Department of Dermatology Universitätsklinikum Erlangen Friedrich‐Alexander‐Universität Erlangen‐Nürnberg Erlangen Germany

**Keywords:** Basal cell carcinoma, melanoma, pharmacology, protein kinase, T‐cell lymphoma

## Abstract

**Background and Objectives:**

Dermatological oral antitumor therapeutics (OAT) are often interaction‐prone and used in complex regimens. The pharmacological/pharmaceutical care program of the randomized AMBORA trial significantly improved medication safety with various OAT; however, dermato‐oncological patients were not included. It was subsequently implemented into clinical routine, including dermato‐oncology. We aimed to analyze medication errors and adherence in patients treated with any dermatological OAT.

**Patients and Methods:**

Medication errors were characterized, for example, according to their cause (PCNE V9.1). Adherence was assessed using the Medication Event Monitoring System (MEMS^®^ Button) and the MARS‐D questionnaire. Primary outcomes were the percentage of resolved OAT‐involving errors and *Dosing Adherence (DA)*; proportion of days with correct OAT intake) over 12 weeks.

**Results:**

In 92 patients (81.5% melanoma), we detected 1.6 medication errors per patient and 61.6% involved the OAT. Thereof, 89.2% were resolved. Of 52 patients participating in the additional adherence monitoring, 48 were evaluable and reached a median *DA* of 95.0% and MARS‐D score of 25/25. *DA* was higher in once‐ vs. twice‐daily regimens (p = 0.0127).

**Conclusions:**

The interprofessional AMBORA care program in dermato‐oncology was associated with the resolution of a large proportion of medication errors and high adherence. Evidence‐based medication management and patient counseling by clinical pharmacologists/pharmacists optimizes medication safety in dermato‐oncological practice.

## INTRODUCTION

Oral antitumor therapeutics (OAT) are increasingly used in various entities, including dermato‐oncology.^1^ The introduction of BRAF and MEK inhibitors has significantly advanced the treatment of melanoma since 2012.^2^ Three BRAF/MEK combination regimens are currently available to treat melanoma.^3^ Moreover, the hedgehog inhibitors sonidegib and vismodegib are available to treat basal cell carcinoma and the retinoid bexarotene represents an oral therapeutic option in cutaneous T‐cell lymphoma.^3^

In general, OAT offer several advantages for patients and treatment teams, such as more convenient administration compared to intravenous therapies.^4^ However, treatment outcomes of OAT can be threatened by inappropriate administration, poor adherence, or potential drug‐drug/drug‐food‐interactions.^1^ Adherence to OAT varies widely, ranging from 14% to 100%, depending on factors such as the patient cohort, dosing regimen, and assessment method.^5^, ^6^ For instance, adherence to imatinib was the only independent predictor of treatment outcomes in chronic myeloid leukemia: patients with adherence below 80% showed no molecular responses.^7^ Dermatological OAT are particularly noteworthy, as they are often used in complex dosing regimens – for example, specific time intervals to food intake for dabrafenib/trametinib or complicated schedules for vemurafenib (twice daily [BID], continuously) and cobimetinib (once daily [OD], cyclic).^8^

The randomized, multicenter AMBORA trial (Medication Safety with Oral Antitumor Therapy, 2017–2020) evaluated an interprofessional care program to optimize medication safety in treatment with various OAT, except for dermato‐oncological patients.^9^, ^10^ In brief, patients were counseled by clinical pharmacologists/pharmacists at four predefined time points over 12 weeks after the initiation of a new OAT. Four key elements were addressed within this care program: *(1)* advanced medication reviews assessing the complete medication, *(2)* structured counseling on the OAT, such as correct drug intake, *(3)* management of side effects, and *(4)* adherence optimization through offering various tools (e.g. apps, diaries).^9^ Medication errors – such as drug‐drug or drug‐food interactions – were frequently identified in the AMBORA trial, with a mean of 1.7 errors per patient within 12 weeks after OAT initiation.^10^ Patient‐reported adherence to OAT was high within the AMBORA trial.^9^

The AMBORA care program was implemented in clinical practice within the AMBORA Competence and Consultation Center (AMBORA Center) at our University Comprehensive Cancer Center, funded by the Deutsche Krebshilfe (German Cancer Aid).^11^ In this process, dermato‐oncological patients treated with OAT were included for the first time. We aimed to *(1)* identify, characterize, and resolve medication errors, *(2)* describe objective and subjective adherence, and *(3)* provide tailored recommendations to optimize medication safety with OAT in dermato‐oncology.

## METHODS

### Study design and patients

This prospective investigation was performed at the AMBORA Center^11^, ^12^ within the University *Comprehensive Cancer Center* Erlangen‐EMN, approved by the ethics committee of the Friedrich‐Alexander‐Universität Erlangen‐Nürnberg, and registered at the German Clinical Trials Register (DRKS00026272).

Ninety‐two dermato‐oncological patients treated with any OAT were counseled by clinical pharmacologists/pharmacists of the AMBORA Center between September 2021 and October 2023 following informed consent. The four elements briefly described above were addressed within the intensified AMBORA care program (advanced medication reviews, structured counseling on the OAT, management of side effects, and adherence optimization).^9^ Counseling was based on standard‐operating‐procedures (SOP) established in the randomized AMBORA trial^9^ and included systematically developed information material for the patients (e.g., antitumor drug fact sheets or information brochures about common side effects). Predefined follow‐up sessions over 12 weeks were offered to all patients who received initial counseling at OAT initiation (≤ week 1). For patients who were first counseled during ongoing OAT treatment, follow‐up sessions were offered on demand only (e.g., in case of side effects).

**FIGURE 1:**
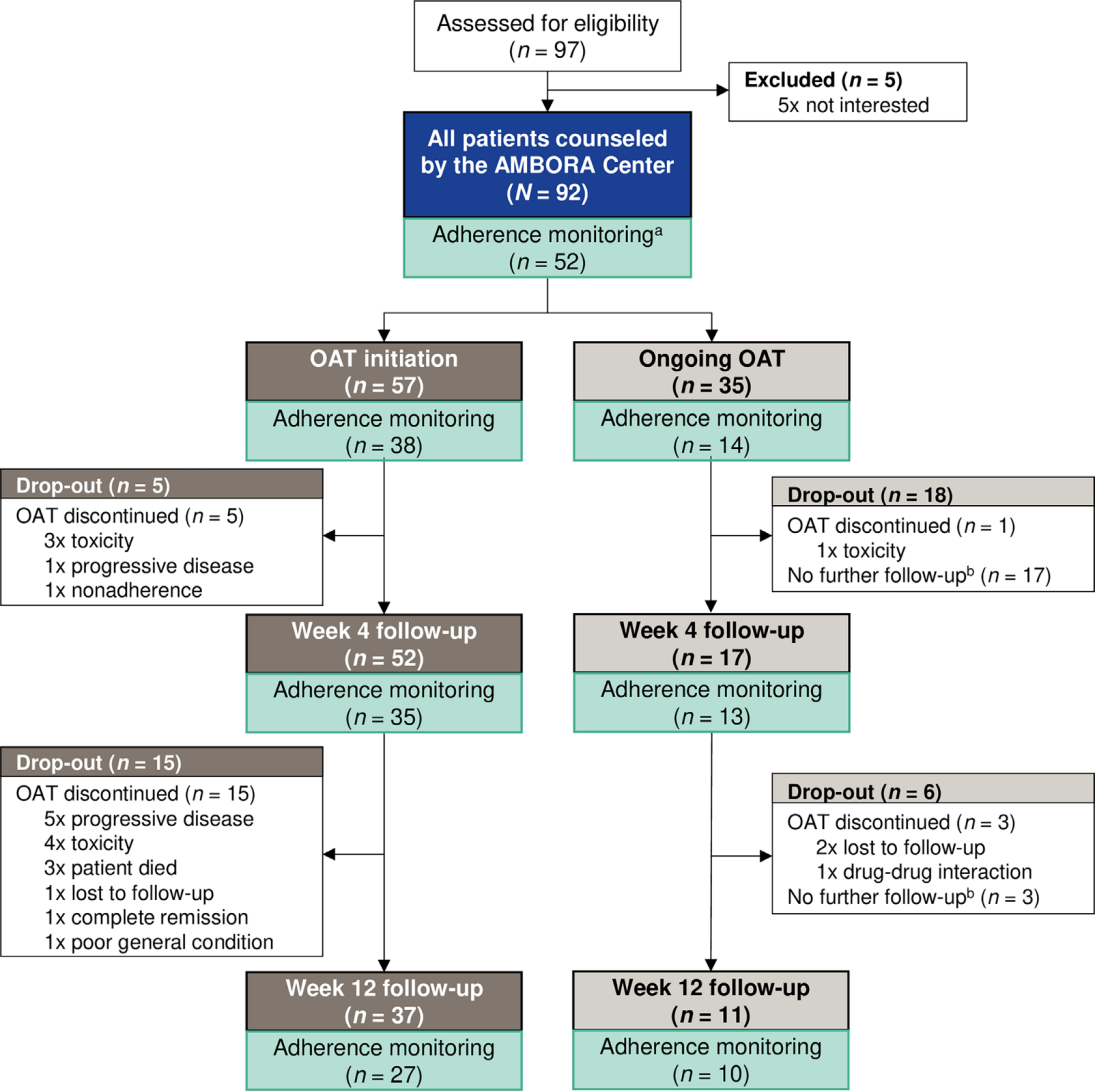
CONSORT diagram of dermato‐oncological patients treated with OAT who were counseled by the AMBORA Center. Patients are stratified for their timepoint of first consultation at therapy initiation or during ongoing therapy and whether they participated in the adherence monitoring. *Abbr*.: OAT, oral antitumor therapeutics. ^a^Forty patients did not participate in the adherence monitoring (33 x participation not offered, 3 x not interested, 2 x linguistic barrier, and 2 x cognitive impairment). ^b^Follow‐up sessions were offered on demand‐only for patients who were first counseled during ongoing OAT (e.g., due to side effects).

We conducted an additional adherence monitoring between October 2022 and October 2023 (Figure 1). Follow‐up sessions were conducted after 4 and 12 weeks for all 52 patients participating in the adherence monitoring at OAT initiation or during ongoing OAT treatment.

**(1) Medication error assessment**

Advanced medication reviews were conducted during all counseling sessions within the AMBORA care program and were based on SOPs established in the AMBORA trial.^9^, ^10^ The complete medication (including the OAT, other prescribed concomitant medications, as well as over‐the‐counter [OTC] drugs and dietary supplements) was thoroughly reviewed. Further details are provided in the online supplementary Methods.

Medication errors were defined according to the National Coordinating Council for Medication Error Reporting and Prevention (NCC MERP) as “any preventable event that may cause or lead to inappropriate medication use or patient harm”.^13^ Medication errors include, for example, drug treatment without an indication, untreated indications, dosage errors, drug‐drug/drug‐food interactions, or administration errors by the patients. Medication errors were classified using validated tools: Pharmaceutical Care Network Europe (PCNE) V9.1^14^ was used to characterize cause and status, whereas severity was assessed using the NCC‐MERP index.^15^ All errors were coded regarding the involved medication (complete medication including the OAT and concomitant medication). For this part, our primary outcome was defined as the reduction of OAT‐involving medication errors over time. Errors were considered resolved if they no longer existed after the implementation of suggested interventions (e.g., stopping or starting a drug, dose modification, or patient education).

**(2) Adherence monitoring**

#### Objective medication adherence

Adherence was objectively monitored using the Medication Event Monitoring System (MEMS^®^) Button^16^ (AARDEX^®^ Group, Seraing, Belgium) over 12 weeks. Patients were instructed to press a separate button once per OAT and timepoint, regardless of the number of tablets or capsules taken or the required time interval to food intake (online supplementary Methods). Adherence parameters were specified in concordance with the ABC taxonomy of medication adherence.^17^ The primary adherence outcome was defined as the number of days with correct numbers of OAT intakes ( = MEMS^®^ Button pressed) related to the observed days (*Dosing Adherence*; *DA*). In line with previously used and internationally well‐established cut‐off values in research on OAT,^7^, ^18^, ^19^ patients were classified as nonadherent if the total *DA* was ≤ 80%. Missing data points without documented causes in patient records or electronic health records (e.g., physician‐ordered interruptions due to side effects) were counted as omitted doses. For instance, treatment interruptions due to fever were not considered as nonadherence, as patients were specifically counseled on this recommended side effect management.

#### Subjective medication adherence

Patient‐reported adherence was subjectively inquired using the validated German MARS‐D questionnaire,^20^ after 4 and 12 weeks. The MARS‐D comprises five questions about medication intake rated on a scale from 1 to 5, leading to a total score of 5 to 25 (25 = fully adherent).^20^

### Statistical analysis

Data were descriptively analyzed using Microsoft Access^®^, Excel^®^, and the MEMS^®^ Adherence software (AARDEX^®^ Group). Statistical testing was based on the intention‐to‐treat principle and performed at 95% confidence intervals using GraphPad Prism^®^. Categorical parameters (e.g., medication error characteristics) were analyzed using two‐sided Chi^2^‐test or Fisher's Exact test. Continuous variables (e.g., adherence data) were compared using parametric t‐test, Mann‐Whitney test, or Wilcoxon rank‐sum test, as appropriate.

## RESULTS

### Patient characteristics

Ninety‐two dermato‐oncological patients treated with nine different OAT were counseled at the AMBORA Center (Table 1). The majority (62.0 %, 57/92) received initial counseling at OAT initiation (Figure 1, CONSORT diagram). At least one follow‐up session was conducted for 76.1 % (70/92) of all patients. No differences were observed in the characteristics of patients counseled at OAT initiation or during ongoing treatment (online supplementary Table S1). The adherence monitoring was offered to 59 patients and 52 (88.1%) consented to take part. Of these, 92.3% (48/52) and 71.5% (37/52) completed the follow‐up at week 4 and week 12, respectively (Figure 1). No differences were observed in characteristics of participants in the adherence monitoring compared to other patients (online supplementary Table S2).

**TABLE 1:** Characteristics of dermato‐oncological patients treated with OAT who were counseled by the AMBORA Center.

Patient characteristics	No. (%) of patients *N* = 92
Age, years (mean, range)	61.1 [29–90]
Female sex	52 (56.5)
ECOG 0–1	66 (71.7)
ECOG > 1	26 (28.3)
Employed/working	22 (23.9)
In need of support	17 (18.5)
Grapefruit consumption	12 (13.0)
Use of ≥ 1 OTC drug^a^	64 (69.6)
**Medication per patient** (median, range)	
All drugs^b^	8 [1‐22]
Oral antitumor therapeutics^c^	2 [1‐3]
Concomitant medication	6 [0‐20]
OTC drugs^a^	1 [0‐10]
**Living situation**	
With partner/family	71 (77.2)
Alone	16 (17.4)
In care institution	4 (4.3)
NA	1 (1.1)
**Tumor type**	
Melanoma	75 (81.5)
Cutaneous T‐cell lymphoma	11 (12.0)
Basal cell carcinoma	6 (6.5)
**Oral antitumor therapeutics** ^d^	
Dabrafenib/trametinib	49 (53.3)
Encorafenib/binimetinib	18 (19.6)
Bexarotene	10 (10.9)
Temozolomide	4 (4.3)
Vemurafenib/cobimetinib	3 (3.3)
Sonidegib	3 (3.3)
Vismodegib	3 (3.3)
Lenvatinib	1 (1.1)
Acitretin	1 (1.1)
**Treatment characteristics**	
Cyclic intake	8 (8.7)
Curative/adjuvant	23 (25.0)
Off‐label^e^	8 (8.7)

*Abbr*.: ECOG, Eastern Cooperative Oncology Group; NA, not applicable; OAT, oral antitumor therapeutics; OTC, over‐the‐counter

*Note*: Characteristics are shown at baseline (timepoint of first consultation). Categorial variables are presented as number (%) of all patients, continuous variables as mean or median [range].

^a^ Includes OTC drugs and dietary supplements.

^b^ Includes drugs of all administration routes (e.g., oral, parenteral, or topical) and OTC drugs, as well as dietary supplements.

^c^ Two patients treated with dabrafenib/trametinib for melanoma were additionally prescribed with exemestane or talazoparib for breast cancer. One patient was treated with bexarotene and methotrexate.

^d^ Only OAT prescribed for the respective dermato‐oncological indications are shown.

^e^ Includes palliative treatment with temozolomide, lenvatinib, acitretin, and adjuvant treatment with encorafenib/binimetinib.

**(1) Medication errors**

#### Numbers of medication errors

**FIGURE 2:**
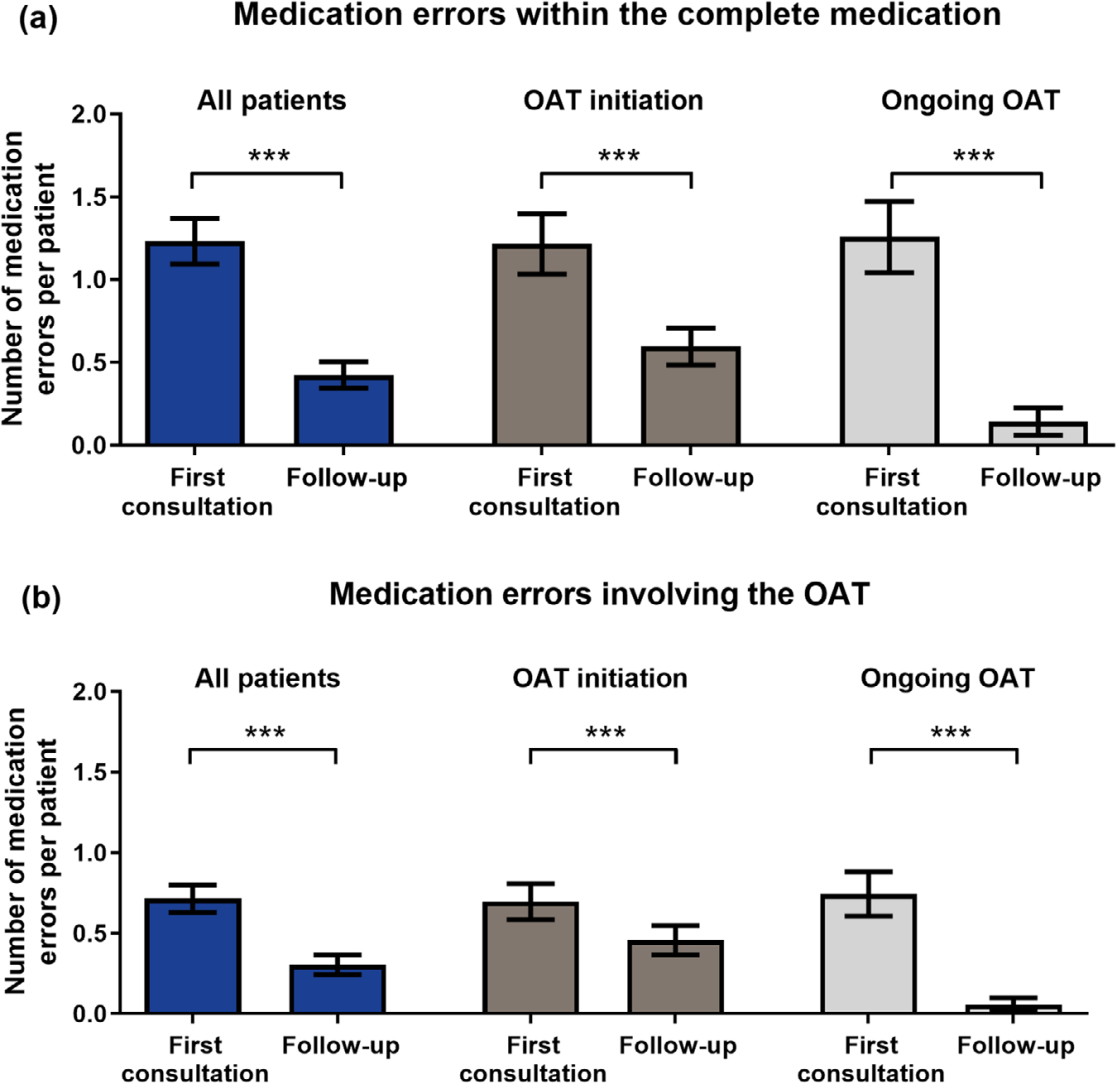
Number of medication errors per patient and the involved medication. Medication errors in all patients, patients with first consultation at OAT initiation, or during ongoing OAT are shown for whether they were (a) detected within the complete medication or (b) the OAT were involved. Medication errors are stratified for the timepoint of their detection (at first consultation or within follow‐up consultations). *Abbr*.: OAT, oral antitumor therapeutics. Data are shown as mean ± standard error of the mean (SEM), ***p < 0.001 (Mann‐Whitney or Wilcoxon rank‐sum test).

Of all patients, 78.3% (72/92) had at least one medication error and most medication errors occurred within the first counseling sessions (Figure 2). We detected 151 errors within the complete medication (OAT and concomitant medication; mean 1.6 per patient, 0–11, from first consultation to last follow‐up) (Figure 2a). Thereof, more medication errors per patient involved the OAT compared to the concomitant medication (mean 1.0, 0–11, vs. 0.6, 0–8, *p* < 0.0006) (Figure 2b). Over time, 87.4% (132/151) of medication errors involving the complete medication and 89.2% (83/93) of OAT‐involving errors were fully resolved.

#### Characteristics of medication errors involving the OAT

**FIGURE 3:**
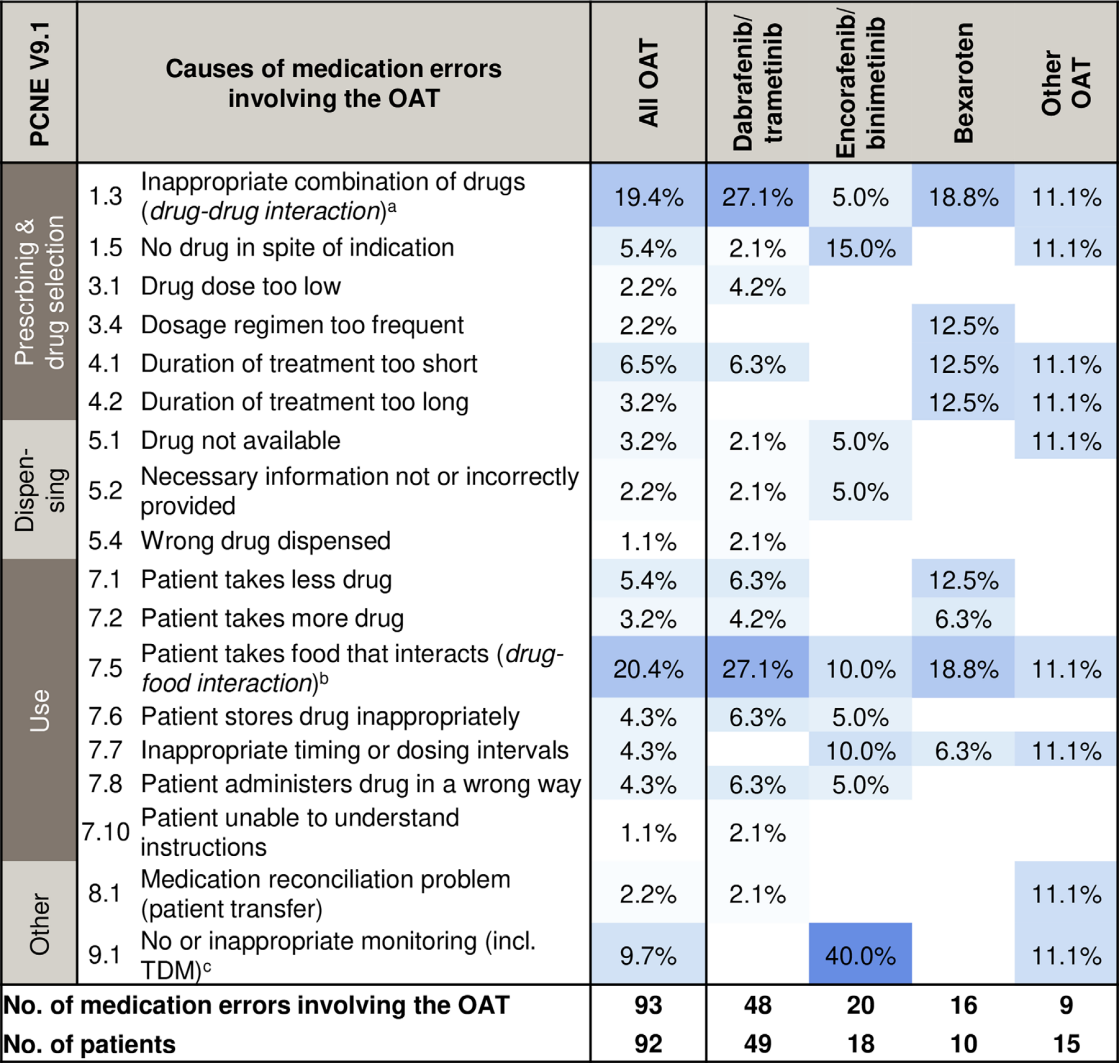
Heatmap of medication errors involving the OAT according to their causes. Medication errors detected in all patients are included and further stratified for the respective OAT regimens. Data are shown as % of medication errors for the respective cause (PCNE V9.1^14^), relative to the number of all medication errors involving the OAT per group. *Abbr*.: CYP, cytochrome P450; OAT, oral antitumor therapeutics; PCNE, Pharmaceutical Care Network Europe; TDM, therapeutic drug monitoring. ^a^For instance, the drug‐drug interaction between dabrafenib (moderate CYP3A4 inducer) and sirolimus (CYP3A4 substrate) was detected in a kidney‐transplanted melanoma patient. Therapeutic drug monitoring was recommended and sirolimus drug levels were outside of the therapeutic range. ^b^Includes over‐the‐counter drugs and dietary supplements. For instance, patients used bexarotene and vitamin A, or dabrafenib and grapefruit juice concomitantly. ^c^For instance, electrocardiography was omitted before re‐initiation of encorafenib/binimetinib in a patient concomitantly treated with amiodarone and current hypokalemia. After intervention, severe QT‐prolongation was diagnosed.

Drug‐drug interactions involving the OAT (19.4%, 18/93) were frequently observed as causes for medication errors (PCNE C1.3) (Figure 3). Selected examples for medication errors involving the OAT attributed to all PCNE causes are provided (online supplementary Table S3). Notably, 43.0% (40/93) of OAT‐involving errors had patient‐related causes (PCNE C7.1–7.10, e.g. nonadherence, inappropriate timing intervals) (Figure 3). Drug‐food interactions involving OTC drugs, dietary supplements, or grapefruit‐products were predominant (20.4%, 19/93). The AMBORA care program prevented 33.3% (31/93) of all OAT‐involving errors from reaching the patients (NCC‐MERP B). Overall, 6.5% (6/93) of OAT‐involving errors were associated with patient harm (NCC‐MERP ≥ E).

**(2) Adherence parameters**

For the primary outcome, patients reached a median objective *Dosing Adherence* (*DA*) of 95.0% (5.4–100.0) (Table 2) over 12 weeks. Patients used the MEMS^®^ Buttons for 71 days (median, 1–121). Subjective, patient‐reported adherence was high (median MARS‐D score 25, 24–25). No differences over time were observed when comparing *DA* and patient‐reported adherence for the first 4 weeks of using the MEMS^®^ Buttons compared to the following 8 weeks (online supplementary Figure S1).

**TABLE 2:** Adherence parameters of all evaluable dermato‐oncological patients who participated in the adherence monitoring.

Adherence parameters	Median [range]
	Evaluable patients *n* = 48
**Objective adherence (MEMS^®^ Buttons)**	
*Dosing Adherence* (%)	
Total	95.0 [5.4–100.0]
OAT OD^a^	96.4 [10.7–100.0]
OAT BID^b^	92.8 [0.0–100.0]
*Taking Adherence* (%)	
Total	97.8 [7.1–103.3]
OAT OD^a^	98.5 [10.7–112.5]
OAT BID^b^	96.2 [3.6–100.0]
*Timing Adherence* (%)	
Total	99.6 [75.0–100.0]
OAT OD^a^	100.0 [66.7–100.0]
OAT BID^b^	100.0 [90.8–100.0]
*Initiation* (days, mean)^c^	0.4 [0.0–5.0]
*Drug Holidays*	
No. per patient	0.0 [0–10]
No. of patients with *≥ 1 Drug Holiday (%)*	19 (39.6)
Duration (days, mean)	1.9 [0.0–30.0]
*Persistence*	
No. of patients with OAT discontinuations (%)	3 (6.3)
Time MEMS^®^ Buttons used (days, mean)	
Total	70.5 [1.0–121.0]
OAT OD^a^	70.0 [1.0–121.0]
OAT BID^b^	77.0 [1.0–121.0]
**Subjective (patient‐reported) adherence**	
MARS‐D total score	25 [24–25]
MARS‐D (%)	100.0 [95.0–100.0]

*Abbr*.: BID, twice‐daily; MARS‐D, Medication Adherence Reporting Scale, validated German translation; MEMS, Medication Event Monitoring System; OAT, oral antitumor therapeutics; OD, once‐daily

*Note*: Categorial variables are presented as number (%) of patients, continuous variables as median [range] unless indicated otherwise. *Dosing Adherence* = number of days with correct number of OAT intakes related to the observed days, *Taking Adherence* = number of intakes related to the prescribed intakes, *Timing Adherence* = proportion of intakes within the predefined time interval of ±3 hours, *Initiation* = time between the first planned and observed intake, *Drug Holidays* = number and duration of omitted intake for at least 48 hours in OAT OD or 24 hours in BID, and *Persistenc*e = unscheduled discontinuations for ≥ 7 days.

^a^ Patients treated with OAT OD: n = 47.

^b^ Patients treated with OAT BID: n = 37.

^c^ Only measured in patients who participated in the adherence monitoring at initiation of OAT: n = 36.

**FIGURE 4:**
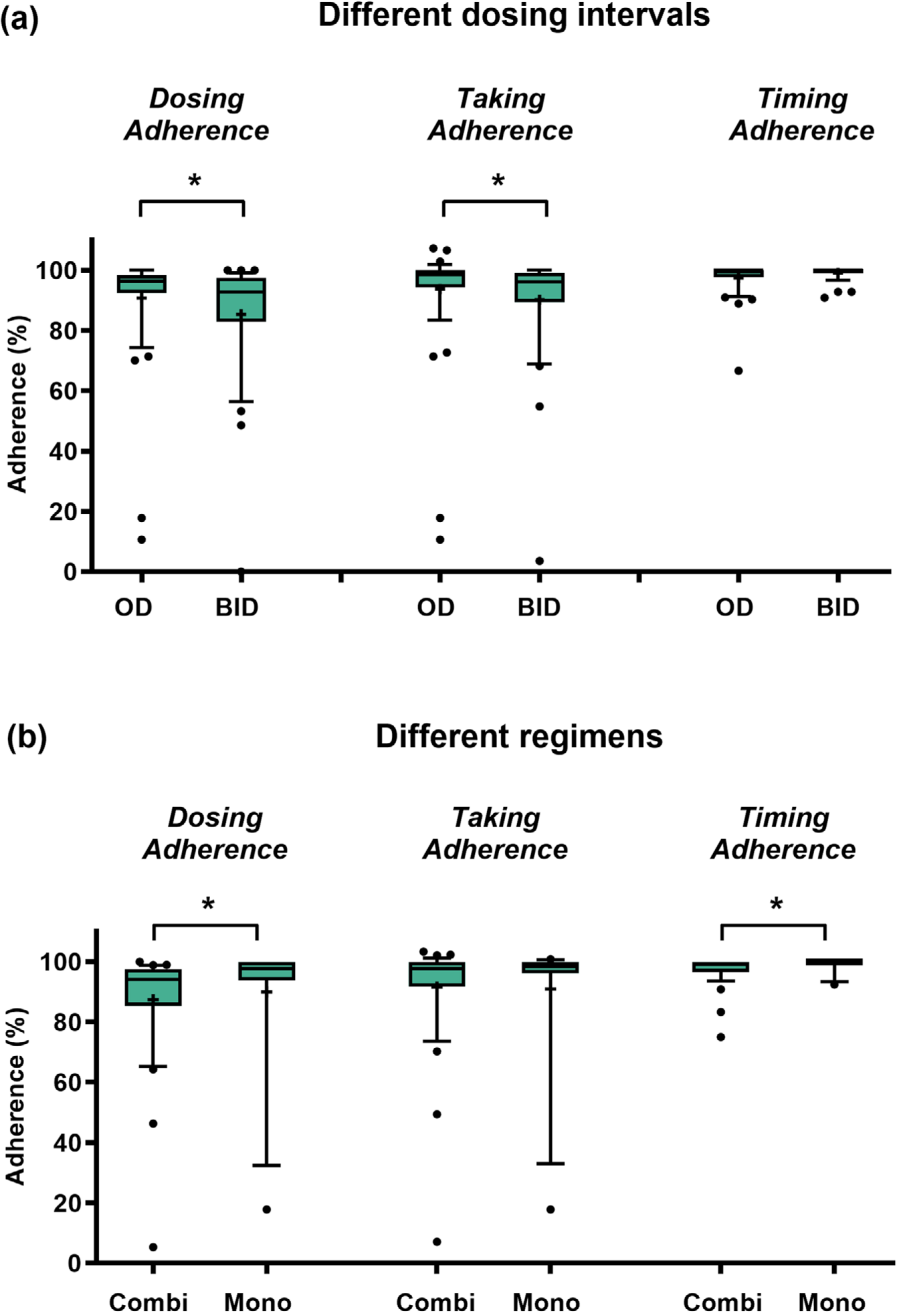
Adherence parameters and the influence of different OAT intake schemes. *Dosing, Taking*, and *Timing Adherence* data are shown for (a) different dosing intervals and (b) different regimens. *Dosing Adherence* = number of days with correct number of OAT intakes related to the observed days, *Taking Adherence* = number of intakes related to the prescribed intakes), and *Timing Adherence* = proportion of intakes within the predefined time interval of ± 3 hours. Data were obtained with MEMS^®^ Buttons and are shown as %. Box‐plots with + representing the mean and whiskers ranging from 10^th^ to 90^th^ percentiles, *p < 0.05 (Mann‐Whitney test). Adherence data for two patients were not evaluable for week 0–12 (1 x patient died and 1 x consent withdrawn, too complicated) and adherence data for six patients were not evaluable for week 4–12 (3 x consent withdrawn, too complicated, 1 x patient died, 1 x lost to follow‐up, and 1 x consent withdrawn, cognitive impairment). *Abbr*.: BID, twice‐daily; Combi; OAT combination; MEMS, Medication Event Monitoring System; Mono, OAT monotherapy; OD, once‐daily; OAT, oral antitumor therapeutics

In total, the median *DA* (96.4% vs. 92.8%, p* = *0.0253) and *Taking Adherence* (98.5% vs. 96.2%, p = 0.0127) were higher in OAT OD compared to OAT BID (Figure 4a). *Dosing*, *Taking*, and *Timing Adherence* neither changed significantly over time in OAT OD nor BID (online supplementary Figure S2). *DA* was significantly higher in patients treated with OAT monotherapies compared to combination regimens (Figure 4b). Adherence parameters stratified for the different OAT are displayed (online supplementary Figure S3a,b). No differences were observed when comparing *Dosing*, *Taking*, and *Timing Adherence* in patients first counseled at OAT initiation vs. during ongoing OAT (online supplementary Figure S3c).

**FIGURE 5:**
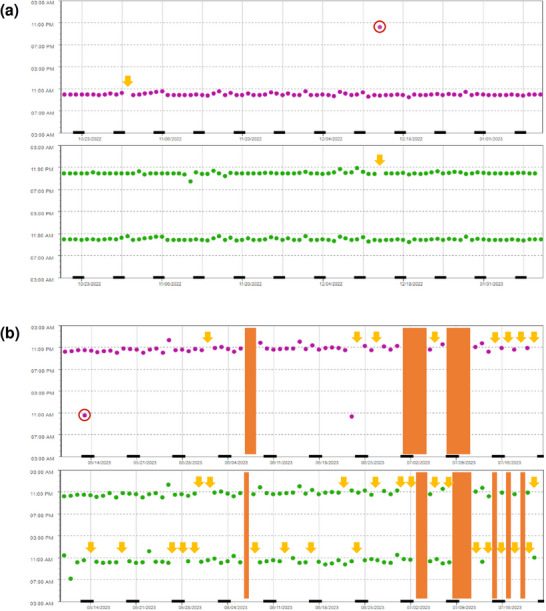
Selected adherence profiles. Examples are shown for (a) an adherent patient and (b) a nonadherent patient. Both patients were treated with a combination of OAT once‐daily (purple dots) and OAT twice‐daily (green dots). Additional OAT intakes by the patient are marked with red circles. Single, omitted OAT intakes are marked with yellow arrows. Multiple, omitted OAT intakes (*Drug Holidays*) are marked with orange bars. Data were obtained with MEMS^®^ Buttons and are shown as date and timepoint of drug intake ( = MEMS^®^ Button pressed). *Abbr*.: MEMS, Medication Event Monitoring System; OAT, oral antitumor therapeutics

Overall, six patients were classified as nonadherent (total *DA* ≤ 80%). Apart from younger mean age (*p* = 0.0008) and a higher proportion of employed/working patients (*p* = 0.0117), no differences were observed between nonadherent and adherent patients (online supplementary Table S4). Examples of adherence profiles are presented in Figure 5a,b.

### Medication safety recommendations

**FIGURE 6:**
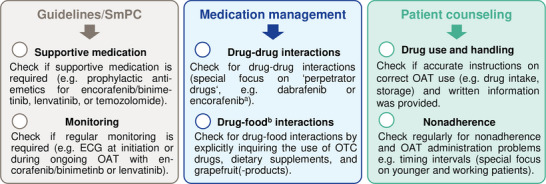
Top recommendations to optimize medication safety with OAT in dermato‐oncological care. *Abbr*.: CYP, cytochrome P450; ECG, electrocardiogram; OAT, oral antitumor therapeutics; OTC, over‐the‐counter; SmPC, summary of product characteristics. ^a^According to new data published after the study period, encorafenib is now categorized as a strong CYP3A4 inducer.^8^ ^b^Includes OTC drugs and dietary supplements.

On the basis of *(1)* the most frequently observed causes of medication errors involving the OAT and *(2)* findings of our adherence monitoring, we compiled a medication safety checklist. These tailored recommendations can help to further optimize medication safety with OAT in dermato‐oncological care (Figure 6).

## DISCUSSION

We performed advanced medication reviews in line with the evidence‐based AMBORA care program^9^ and assessed objective as well as subjective adherence to dermatological oral antitumor therapeutics using the Medication Event Monitoring System (MEMS^®^) Button and the MARS‐D questionnaire. First, we detected a high number of 1.6 medication errors per patient within the complete medication, of which nearly two‐thirds involved the OAT (Figure 2) and 89% were resolved. Second, *Dosing Adherence* (*DA*) to the OAT was high over 12 weeks (95%).

Drug‐drug/drug‐food interactions were the most common causes of OAT‐involving errors (approximately 40%) (Figure 3). Data from patients with other entities counseled within our AMBORA Center showed that drug‐drug/drug‐food interactions accounted for 24% of errors,^21^ thereby supporting the notion that dermatological OAT are particularly interaction‐prone. Another study in patients with various entities indicated that drug‐drug interactions involving the OAT accounted for 14% of potential interactions.^22^ These data are only comparable to a limited extent due to heterogeneous assessment methods, but indicate that targeted strategies are required to address these challenges. For instance, the German Summary of Product Characteristics (SmPC) of dabrafenib recommends to perform a critical ‘drug utilization review’ prior to initiation.^8^ While the potent cytochrome P450 (CYP) 3A4 induction by dabrafenib has been well‐known for years, a similar or even stronger interaction potential for encorafenib was reported by new data published after our investigation was completed.^8^

We observed a high percentage of patients using OTC drugs including dietary supplements (70%). Surveys in melanoma patients showed that 27% of 100 participants reported using complementary and alternative medicine (CAM), while only 50% informed their physicians.^23^ Hence, CAM use should be explicitly inquired about prior to therapy initiation. In an interprofessional approach, oncology pharmacists play a key role in addressing drug‐drug and drug‐food interactions^24^ as well as side effects.^25^ These data underline the need for thorough medication reviews, including OTC drugs and dietary supplements (e.g., CAM), as one of our key recommendations to optimize medication safety (Figure 6).

Overall, 43% of medication errors involving the OAT had patient‐related causes. Among other interventions, counseling by clinical pharmacists, patient education, and medication management are considered successful and practical for promoting adherence,^26^ and involving pharmacists within the treatment teams is encouraged by multiple investigations.^27^, ^28^

Overall, the median objective (95%, *DA*) and subjective (100%) adherence in patients counseled a our AMBORA Center are clearly within the upper range of literature data.^5^, ^6^ However, data on other OAT are difficult to compare as assessment methods vary and only few studies used both objective and subjective methods. An investigation of patients with solid tumors (gastrointestinal and breast cancer) and hemato‐oncological patients within a pharmacist‐led OAT management program over 12 weeks detected medication possession ratios of 85% and 94%, and subjective adherence of 86% and 95% for the respective populations.^29^ Marin et al. used MEMS^®^ Caps, self‐reporting, and pill count to measure adherence in patients treated with imatinib once‐daily: After about 3 months, median adherence measured by MEMS^®^ Caps was 98% and 14% of patients had adherence rates ≤ 80%.^7^ Self‐reports and pill counts overestimated adherence.^7^ Another study measured adherence to capecitabine in breast and colorectal patients within a pharmaceutical care program over approximately 4 months using MEMS^®^ Caps and found a comparable, mean *DA* of 97%.^30^

In line with other publications,^31^, ^32^ we observed lower adherence in twice‐daily OAT regimens compared to once‐daily (Figure 4). Given the predominant use of combination therapies with BRAF/MEK inhibitors, melanoma patients could be considered particularly susceptible to nonadherence. Despite the intensified AMBORA care program, six patients were identified as being nonadherent (e.g., Figure 5b). We found that adherence can be a particular problem for younger patients, an observation that has previously been reported e.g., in breast cancer^33^ or in psoriasis‐patients on systemic treatment^34^. Whereas diagnosis and subsequent treatment with OAT tends to be limited to advanced age in basal cell carcinoma or cutaneous T‐cell lymphoma, OAT for melanoma are increasingly used in earlier treatment lines including in younger patients (e.g., adjuvant therapies).^35^ In a systematic review, adherence in adjuvant hormonal breast cancer therapy ranged from 41–72% after 5 years, with 31–73% treatment discontinuations.^36^ Nonadherence and discontinuations were associated with increased mortality in this setting.^37^ Thus, individual circumstances (e.g., age, working life) should be specifically addressed in interprofessional care programs (Figure 6).

We consider the combined assessment of objective and subjective adherence as a major strength of our investigation. While validated patient‐reported questionnaires are a common and practical way of identifying nonadherence, their reliability is often inconclusive and they might overestimate adherence compared to objective methods.^7^, ^26^ Furthermore, our care program was based on SOP established in the AMBORA trial^9^ to ensure validity. By counseling patients and offering adherence monitoring in routine dermato‐oncological care, we were able to include patients regardless of treatment duration and consider the participants in the adherence monitoring as a representative subgroup (online supplementary Table S3). Dermato‐oncological patients are often simultaneously treated with a combination of once‐daily and twice‐daily OAT. Investigating adherence in this cohort allowed us to compare adherence between once‐daily and twice‐daily OAT in the same patient, thereby eliminating any patient‐specific factors that might influence adherence.

We acknowledge some limitations of our work: For ethical reasons, we performed a nonrandomized investigation of the AMBORA care program in dermato‐oncology and set out to describe adherence in this cohort. We would expect medication safety or adherence to be lower in clinical routine without the AMBORA care program. Using the MEMS^®^ Buttons was only a surrogate for drug intake and we could not assess the actual numbers of tablets/capsules taken or any time interval between drug and food intake. On the one hand, this might have underestimated adherence. Therapeutic drug monitoring^38^, ^39^ could be performed to outweigh this bias, but its informative value would still be limited as patients may only have adhered to the OAT on the days before blood sampling. On the other hand, the intervention itself might have promoted adherence, particularly within the first weeks. Interestingly, we observed no differences when comparing adherence rates over time (online supplementary Figures S1, S2). Moreover, nonadherence could not be addressed within the study period, as adherence profiles were only retrospectively evaluated. Future work should include proactive counseling based on adherence profiles, similar to a recent study in patients treated with various OAT.^40^

Taken together, medication errors (e.g., drug‐drug/drug‐food interactions) were frequent in treatment with dermatological OAT and the majority was resolved within the AMBORA Center. Both objective and subjective adherence to the OAT was high. Special focus should be given to adherence in twice‐daily OAT regimens and in younger, still employed/working patients. Our data highlights the benefit of interprofessional collaboration involving clinical pharmacologists/pharmacists. We provide tailored recommendations to optimize medication safety and adherence in dermato‐oncological patients treated with OAT.

## FUNDING

The work was supported by the German Cancer Aid (Deutsche Krebshilfe; project grant number 70114066/70114067). The funding sources had no influence on its design, data collection, analysis, or interpretation.

## CONFLICT OF INTEREST STATEMENT

L.C. declares no conflict of interest. F.D. has received consulting fees from Lilly and Sandoz‐Hexal; honoraria from Johnson & Johnson and Gilead; and reports other financial or non‐financial interests (earmarked financial contribution: first award of the MSD Germany Health Award 2021). R.K. has received honoraria from Pierre Fabre and support for meetings/travel from Pierre Fabre, Novartis, and SUN Pharmaceuticals. P.D. has received honoraria from AstraZeneca GmbH and reports other financial or non‐financial interests (earmarked financial contribution: first award of the MSD Germany Health Award 2021). M.E. has received honoraria from Immunocore, Novartis, Pierre Fabre, and Sanofi; support for meetings/travel from Novartis and Pierre Fabre; and served on a Data Safety Monitoring Board/Advisory Board for Sanofi. M.F.F. has received grants or contracts from Boehringer Ingelheim and Heidelberg Pharma Research GmbH; consulting fees and honoraria from Boehringer Ingelheim; and reports other financial or non‐financial interests (earmarked financial contribution: first award of the MSD Germany Health Award 2021). C.B. has received consulting fees from Almirall Hermal, BMS, Delcath, Immunocore, MSD, Novartis, Pierre Fabre, Regeneron, and Sanofi; honoraria from Almirall Hermal, BMS, Leo Pharma, MSD, Novartis, and Pierre Fabre; support for meetings/travel from Pierre Fabre; and served on a Data Safety Monitoring Board/Advisory Board for Miltenyi and InflaRx. K.G. has received honoraria from AstraZeneca GmbH and Roche Pharma and reports other financial or non‐financial interests (earmarked financial contribution: first award of the MSD Germany Health Award 2021).

## Supporting information



Supplementary information
