# Supplementary material for: Implications for medication safety and adherence in dermato‐oncology: The AMBORA care program for oral antitumor therapeutics
Source: J Dtsch Dermatol Ges. 2025 Jul 17;23(10):1245–56. doi: 10.1111/ddg.15809 (PMC12548322; doi:10.1111/ddg.15809)
Supplement: Supplementary file 1 — Supplementary information [file DDG-23-1245-s001.pdf]

ONLINE SUPPLEMENTARY INFORMATION

Cuba L et al. Implications for Medication Safety and Adherence in Dermato-Oncology: The AMBORA Care Program for Oral Antitumor Therapeutics

ONLINE SUPPLEMENTARY METHODS **2**
ONLINE SUPPLEMENTARY FIGURES **4**
FIGURE S1 *Dosing Adherence* and patient-reported adherence over time. 4
FIGURE S2 *Dosing*, *Taking*, and *Timing Adherence* for different OAT dosing intervals over time. 5
FIGURE S3 *Dosing*, *Taking*, and *Timing Adherence* in different OAT regimens or timepoint of treatment. 6
ONLINE SUPPLEMENTARY TABLES **8**
TABLE S1 Characteristics of dermato-oncological patients treated with OAT separately presented for patients who were first counseled at OAT initiation and during ongoing treatment. 8
TABLE S2 Characteristics of dermato-oncological patients treated with OAT who were counseled by the AMBORA Center separately presented for patients who participated in the adherence monitoring or not. 10
TABLE S3 Selected examples of medication errors involving the OAT detected in dermato-oncological patients. 12
TABLE S4 Characteristics of dermato-oncological patients treated with OAT separately presented for adherent patients (total *Dosing Adherence* >80%) and nonadherent patients (total *Dosing Adherence* ≤80%). 14

ONLINE SUPPLEMENTARY METHODS

(1) Medication error assessment

Advanced medication reviews were performed according to the Pharmaceutical Care Network Europe (PCNE).[1] Summaries of product characteristics (SmPC) and other evidence-based sources were considered to evaluate medication appropriateness: for instance, databases for drug-drug/drug-food interactions,[2-4] or guidelines for supportive medication (e.g. antiemesis,[5]).

(2) Adherence monitoring

The MEMS® Buttons,[6] did not actively remind patients to take the OAT. A separate button for each OAT was handed out after instruction (i.e. two buttons for combination therapies) and could be attached to the packaging. Timepoints of medication intake were recorded and retrospectively analyzed after return of the buttons. A form was provided to document technical problems or intake errors (e.g. correct OAT intake, but pressing the buttons forgotten). These events were not counted as omitted intakes.

Adherence parameters

- *Dosing Adherence* = number of days with correct number of OAT intakes related to the observed days
- *Taking Adherence* = number of intakes related to the prescribed intakes)
- *Timing Adherence* = proportion of intakes within the predefined time interval of ±3 hours
- *Initiation* (I, time between the first planned and observed intake)
- *Drug Holidays* (number and duration of omitted intake for at least 48 hours in OAT OD or 24 hours in BID)
- *Persistence* (unscheduled discontinuations for ≥7 days)

References

1. Pharmaceutical Care Network Europe (PCNE) position paper on medication review (2016). Available from: https://www.pcne.org/upload/files/149_Position_Paper_on_PCNE_Medication_Review_final.pdf (last access on 30.01.2024).
2. UpToDate®. Drug interactions. Available from: https://www.uptodate.com/contents/search (last access on 30.01.2024).
3. Pharmaceutical press: Stockley's interactions checker. Available from: https://www.medicinescomplete.com/log-in/ (last access on 30.01.2024).
4. Memorial Sloan Kettering Cancer Center (MSKCC): Search about herbs. Available from: https://www.mskcc.org/cancer-care/diagnosis-treatment/symptom-management/integrative-medicine/herbs/search (last access on 30.01.2024).
5. National Comprehensive Cancer Network (NCCN) clinical practice guidelines in oncology: Antiemesis. Version 1.2024, December 13, 2023. Available from: https://www.nccn.org/professionals/physician_gls/pdf/antiemesis.pdf (last access on 04.06.2024).
6. AARDEX® Group. MEMS® Button ('Medication Event Monitoring System'). Available from: https://aardexgroup.com/medication-event-monitoring-system/ (last access on 04.06.2024)

ONLINE SUPPLEMENTARY FIGURES

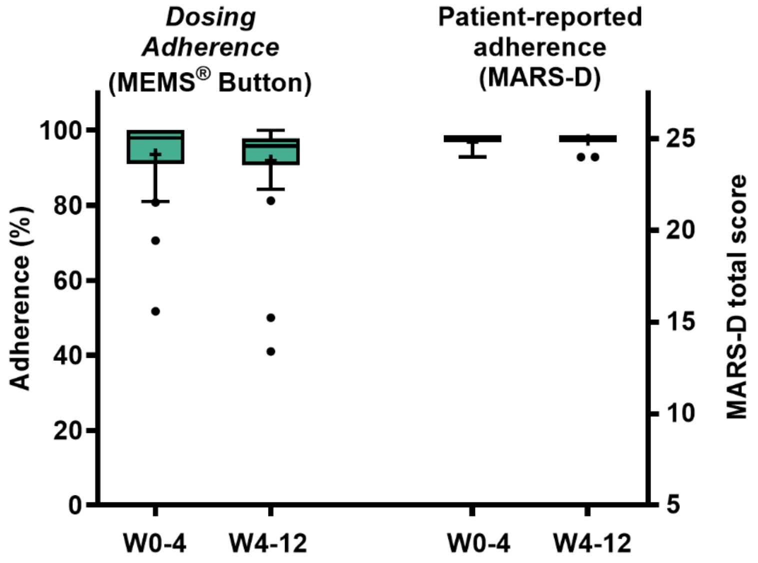

FIGURE S1 *Dosing Adherence* and patient-reported adherence over time**.**

Data are stratified for week 0 to 4 and week 4 to 12 of participating in the adherence monitoring. *Dosing Adherence* was measured with MEMS® Buttons and is expressed as %. Patient-reported adherence was measured with MARS-D and is shown as % of total score (5-25). Box-plots with + representing the mean and whiskers ranging from 10th to 90th percentiles, Wilcoxon matched paired test.

MARS-D, Medication Adherence Reporting Scale, validated German translation; MEMS, Medication Event Monitoring System; W, week.

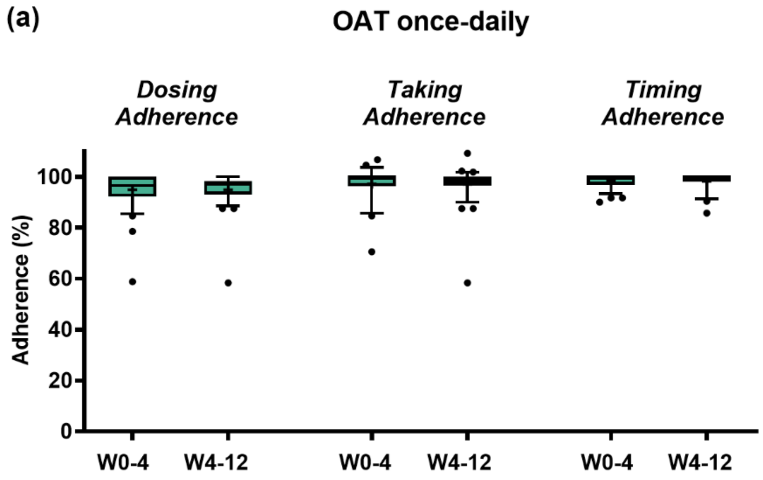

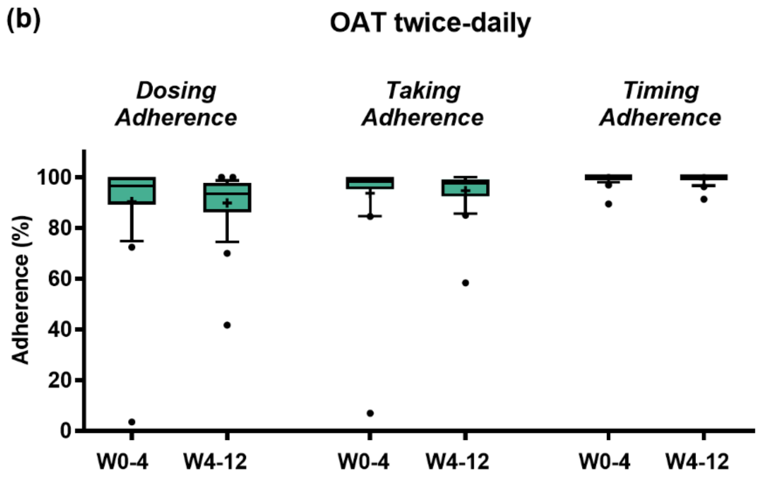

FIGURE S2 *Dosing*, *Taking*, and *Timing Adherence* for different OAT dosing intervals over time.

Data are shown for **(a)** patients treated with OAT once-daily and **(b)** OAT twice-daily. Parameters are stratified for week 0 to 4 and week 4 to 12 of participating in the adherence monitoring. Data were measured with MEMS® Buttons and are expressed as %. Box-plots with + representing the mean and whiskers ranging from 10th to 90th percentiles, Wilcoxon matched paired test.

MEMS, Medication Event Monitoring System; OAT, oral antitumor therapeutics; W, week.

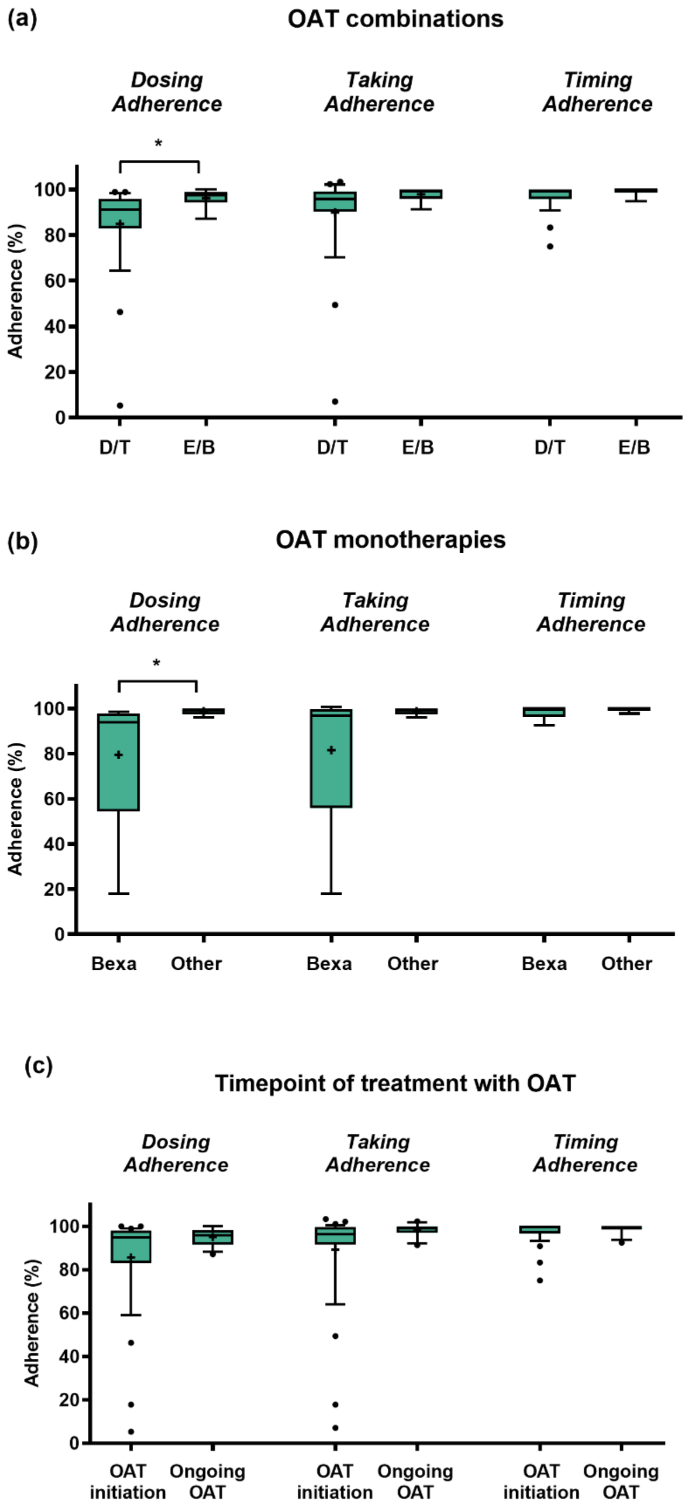

FIGURE S3 *Dosing*, *Taking*, and *Timing Adherence* in different OAT regimens or timepoint of treatment.

Data are shown for patients treated with **(a)** different OAT combinations, **(b)** different OAT monotherapies, and **(c)** patients who were first counseled at OAT initiation or during ongoing OAT. Data were measured with MEMS® Buttons and are expressed

as %. Box-plots with + representing the mean and whiskers ranging from 10th to 90th percentiles, *$p < .05$ (Mann-Whitney test).

Bexa, bexarotene; D/T, dabrafenib/trametinib; E/B, encorafenib/binimetinib; MEMS, Medication Event Monitoring System; OAT, oral antitumor therapeutics.

ONLINE SUPPLEMENTARY TABLES

TABLE S1 Characteristics of dermato-oncological patients treated with OAT separately presented for patients who were first counseled at OAT initiation and during ongoing treatment.

	No. (%) of patients		
	**OAT initiation** ***n*=57**	**Ongoing OAT** ***n*=35**	***p***
**Patient characteristics**			
Age, years (mean)	62.4 [29-90]	59.0 [31– 88]	*ns*
Female sex	34 (59.6)	18 (51.4)	*ns*
ECOG 0-1	39 (68.4)	27 (77.1)	*ns*
ECOG >1	18 (31.6)	8 (22.9)	
Employed/working	12 (21.1)	10 (28.6)	*ns*
In need of support	11 (19.3)	6 (17.1)	*ns*
Grapefruit consumption	8 (14.0)	4 (11.4)	*ns*
Use of ≥1 OTC drug[a]	39 (68.4)	25 (71.4)	*ns*
**Medication per patient** (median)			
All drugs[b]	9 [1-21]	7 [3-22]	*ns*
Oral antitumor therapeutics[c]	2 [1-3]	2 [1-3]	*ns*
Concomitant medication	7 [0-20]	6 [1-20]	*ns*
OTC drugs[a]	1 [0-10]	1 [0-5]	*ns*
**Living situation**			
With partner/family	43 (75.4)	28 (80.0)	*ns*
Alone	11 (19.3)	5 (14.3)	
In care institution	3 (5.3)	1 (2.9)	
NA	–	1 (2.9)	
**Tumor type**			
Melanoma	48 (84.2)	27 (77.1)	*ns*
Cutaneous T-cell lymphoma	6 (10.5)	5 (14.3)	
Basal cell carcinoma	3 (5.3)	3 (8.6)	
**Oral antitumor therapeutics[d]**			
Dabrafenib/trametinib	35 (61.4)	14 (40.0)	*ns*
Encorafenib/binimetinib	7 (12.3)	11 (31.4)	
Bexarotene	5 (8.8)	5 (14.3)	
Temozolomide	3 (5.3)	1 (2.9)	
Vemurafenib/cobimetinib	2 (3.5)	1 (2.9)	
Sonidegib	1 (1.8)	2 (5.7)	
Vismodegib	2 (3.5)	1 (2.9)	
Lenvatinib	1 (1.8)	–	
Acitretin	1 (1.8)	–	
**Treatment characteristics**			
Cyclic intake	5 (8.8)	3 (8.6)	*ns*
Curative/adjuvant	16 (28.1)	7 (20.0)	*ns*
Off-label[e]	5 (8.8)	3 (8.6)	*ns*

Abbreviations: ECOG, Eastern Cooperative Oncology Group; NA, not applicable; OAT, oral antitumor therapeutics; OTC, over-the-counter.

Note: Characteristics are shown at baseline (timepoint of first consultation). Categorial variables are presented as number (%) of patients per group, continuous variables as mean or median [range]. ns, not significant (t-test, Mann-Whitney, Chi^2, or Fishers' Exact test).

[a]Includes OTC drugs and dietary supplements.

[b]Includes drugs of all administration routes (e.g. oral, parenteral, or topical) and OTC drugs, as well as dietary supplements.

[c]Two patients treated with dabrafenib/trametinib for melanoma were additionally prescribed with exemestane and talazoparib for breast cancer. One patient was treated with bexarotene and methotrexate.

[d]Only OAT prescribed for dermato-oncological indications are shown.

[e]Includes palliative treatment with temozolomide, lenvatinib, acitretin, and adjuvant treatment with encorafenib/binimetinib.

TABLE S2 Characteristics of dermato-oncological patients treated with OAT who were counseled by the AMBORA Center separately presented for patients who participated in the adherence monitoring or not.

	No. (%) of patients		
	Participating in adherence monitoring *n*=52	Others *n*=40	*p*
**Patient characteristics**			
Age, years (mean)	61.6 [29-90]	60.4 [31-88]	*ns*
Female sex	29 (55.8)	23 (57.5)	*ns*
ECOG 0-1	37 (71.2)	29 (72.5)	*ns*
ECOG >1	15 (28.8)	11 (27.5)	
Employed/working	13 (25.0)	9 (22.5)	*ns*
In need of support	10 (19.2)	7 (17.5)	*ns*
Grapefruit consumption	9 (17.3)	3 (7.5)	*ns*
Use of ≥1 OTC drug[a]	38 (73.1)	26 (65.0)	*ns*
**Medication per patient** (median)			
All drugs[b]	9 [1-16]	7 [2-22]	*ns*
Oral antitumor therapeutics[c]	2 [1-2]	2 [1-3]	*ns*
Concomitant medication	7 [0-15]	6 [0-20]	*ns*
OTC drugs[a]	1 [0-9]	1 [0-10]	*ns*
**Living situation**			
With partner/family	43 (82.7)	28 (70.0)	*ns*
Alone	8 (15.4)	8 (20.0)	
In care institution	1 (1.9)	3 (7.5)	
NA	–	1 (2.5)	
**Tumor type**			
Melanoma	44 (84.6)	31 (77.5)	*ns*
Cutaneous T-cell lymphoma	6 (11.5)	5 (12.5)	
Basal cell carcinoma	2 (3.8)	4 (10.0)	
**Oral antitumor therapeutics[d]**			
Dabrafenib/trametinib	32 (61.5)	17 (42.5)	*ns*
Encorafenib/binimetinib	9 (17.3)	9 (22.5)	
Bexarotene	5 (9.6)	5 (12.5)	
Temozolomide	2 (3.8)	2 (5.0)	
Vemurafenib/cobimetinib	–	3 (7.5)	
Sonidegib	1 (1.9)	2 (5.0)	
Vismodegib	1 (1.9)	2 (5.0)	
Lenvatinib	1 (1.9)	–	
Acitretin	1 (1.9)	–	
**Treatment characteristics**			
Cyclic intake	2 (3.8)	6 (15.0)	*ns*
Curative/adjuvant	13 (25.0)	13 (32.5)	*ns*
Off-label[e]	5 (9.6)	3 (7.5)	*ns*

Abbreviations: ECOG, Eastern Cooperative Oncology Group; NA, not applicable; OAT, oral antitumor therapeutics; OTC, over-the-counter. *Note:* Characteristics are

shown at baseline (timepoint of first consultation). Categorial variables are presented as number (%) of patients per group, continuous variables as mean or median [range]. ns, not significant (t-test, Mann-Whitney, Chi^2, or Fishers' Exact test).

[a]Includes OTC drugs and dietary supplements.

[b]Includes drugs of all administration routes (e.g. oral, parenteral, or topical) and OTC drugs, as well as dietary supplements.

[c]Two patients treated with dabrafenib/trametinib for melanoma were additionally prescribed with exemestane and talazoparib for breast cancer. One patient was treated with bexarotene and methotrexate.

[d]Only OAT prescribed for dermato-oncological indications are shown.

[e]Includes palliative treatment with temozolomide, lenvatinib, acitretin, and adjuvant treatment with encorafenib/binimetinib.

TABLE S3 Selected examples of medication errors involving the OAT detected in dermato-oncological patients.

Cause	Causes of medication errors		Selected examples
Prescrbinig & drug selection	1.3	Inappropriate combination of drugs (*drug-drug interaction*)	**Dabrafenib** (moderate CYP3A4 inducer) → exemestane↓, simvastatin↓, or sirolimus↓;<br>**bexarotene** (moderate CYP3A4 inducer) → ↓ atorvastatin
	1.5	No drug in spite of indication	Prophylactic antiemetic treatment omitted in OAT with moderate/high emetogenic risk (e.g. **encorafenib/binimetinib** or **temozolomide**)
	3.1	Drug dose too low	Dose of **dabrafenib/trametinib** 50% too low in a melanoma patient with brain metastases and high symptomatic burden
	3.4	Dosage regimen too frequent	**Bexarotene** prescribed twice or four times per day instead of once-daily (risk for nonadherence ↑)
	4.1	Duration of treatment too short	Refill prescriptions for **dabrafenib/trametinib**, **bexarotene**, or **vismodegib** not provided in time
	4.2	Duration of treatment too long	**Lenvatinib** not temporarily discontinued prior to elective surgery;<br>supportive medication (e.g. fenofibrate) not stopped at permanent discontinuation of **bexarotene**
Dispensing	5.1	Drug not available	Retail pharmacy unable to order **dabrafenib/trametinib** or **sonidegib**
	5.2	Necessary information not or incorrectly provided	Intake instructions for **encorafenib/binimetinib** not provided by health care professionals
	5.4	Wrong drug dispensed	**Dabrafenib** and **trametinib** mixed up in preparation of drugs by nursing staff
Use	7.1	Patient takes less drug	Nonadherence (e.g. treatment discontinuation of **bexarotene** or dose reductions of **dabrafenib/trametinib)** without consulting a physician
	7.2	Patient takes more drug	**Trametinib** intake twice-daily instead of once-daily
	7.5	Patient takes food that interacts (*drug-food interaction*)[a]	Grapefruit (moderate CYP3A4 inhibitor) → ↑ **dabrafenib,** ↑ **encorafenib**; **bexarotene** (retinoid) ↔ vitamin A;<br>**vemurafenib** (moderate CYP1A2 inhibitor) → ↑ caffeine
	7.6	Patient stores drug inappropriately	**Dabrafenib** stored in the refrigerator instead of at room temperature; hand washing omitted after handling with **encorafenib/binimetinib** with grandchildren at home
	7.7	Inappropriate timing or dosing intervals	Wrong time interval to food intake for **sonidegib** (1 hour after food/2 hours before food instead of 1 hour before/2 hours after food);<br>dose timing intervals of **binimetinib** after 8/16 hours instead of every 12 hours
	7.8	Patient administers drug in a wrong way	Drug intake of **dabrafenib/trametinib** with food instead of fasting; drinking of one glass of water per tablet/capsule of **encorafenib/binimetinib**
	7.10	Patient unable to understand instructions	Melanoma patient treated with **dabrafenib/trametinib** unaware of sun protection for the skin despite multiple educational measures by physicians/pharmacists
Other	8.1	Medication reconciliation problem (patient transfer)	Documented **dabrafenib** dosage of 75mg 2-0-2, but patient takes 1-0-1 due to kidney impairment; documented intake schedule of **temozolomide** on days 1-5 followed by 21 days of treatment interruption instead of 23 days
	9.1	No or inappropriate monitoring (incl. TDM)	ECG omitted prior to initiation or during ongoing therapy with **encorafenib/binimetinib** or **lenvatinib**

Abbreviations: ↑, increased toxicity; ↓, decreased effectiveness; →, perpetrator drug → victim drug; ↔, additive effect; CYP, cytochrome P450 enzyme; ECG, electrocardiogram; OAT, oral antitumor therapeutics; PCNE, Pharmaceutical Care Network Europe; TDM, therapeutic drug monitoring.

Note: OAT are shown in bold.

[a]Includes over-the-counter drugs and dietary supplements.

TABLE S4 Characteristics of dermato-oncological patients treated with OAT separately presented for adherent patients (total *Dosing Adherence* >80%) and nonadherent patients (total *Dosing Adherence* ≤80%).

	No. (%) of patients		
	**Adherent** *n*=42	**Nonadherent** *n*=6	***p***
**Patient characteristics**			
Age, years (mean)	64.2 [31-90]	44.5 [29-59]	***
Female sex	24 (57.1)	4 (66.7)	*ns*
ECOG 0-1	29 (69.0)	6 (100.0)	*ns*
ECOG >1	13 (31.0)	–	
Grapefruit consumption	7 (16.7)	1 (16.7)	*ns*
Use of ≥1 OTC drug[a]	31 (73.8)	5 (83.3)	*ns*
Employed/working	8 (19.0)	4 (66.7)	*
In need of support	8 (19.0)	–	*ns*
**Medication per patient** (median)			
All drugs[b]	9 [1-16]	7 [4-11]	*ns*
Oral antitumor therapeutics[b]	2 [1-3]	2 [1-2]	*ns*
Concomitant medication	8 [0-15]	5 [2-10]	*ns*
OTC drugs[a]	1 [0-9]	3 [0-7]	*ns*
**Living situation**			
With partner/family	33 (78.6)	6 (100.0)	*ns*
Alone	8 (19.0)	–	
In care institution	1 (2.4)	–	
**Tumor type**			
Melanoma	35 (83.3)	5 (83.3)	*ns*
Cutaneous T-cell lymphoma	5 (11.9)	1 (16.7)	
Basal cell carcinoma	2 (4.8)	–	
**Oral antitumor therapeutics[d]**			
Dabrafenib/trametinib	24 (57.1)	5 (83.3)	*ns*
Bexarotene	4 (9.5)	1 (16.7)	
Others	14 (33.3)	–	
**Treatment characteristics**			
Cyclic intake	2 (4.8)	–	*ns*
Curative/adjuvant	9 (21.4)	3 (50.0)	*ns*
Off-label[e]	5 (11.9)	–	*ns*
OAT initiation (<7 days)	30 (71.4)	6 (100.0)	*ns*

Abbreviations: ECOG, Eastern Cooperative Oncology Group; NA, not applicable; OAT, oral antitumor therapeutics; OTC, over-the-counter. *Note:* Categorial variables are presented as number (%) of patients per group, continuous variables as mean or median [range].

*p < .05; ***p < .001; ns, not significant (t-test, Mann-Whitney, Chi^2, or Fishers' Exact test).

[a]Includes OTC drugs and dietary supplements.

[b]Includes drugs of all administration routes (e.g. oral, parenteral, or topical) and OTC drugs, as well as dietary supplements.

[c]One patient treated with dabrafenib/trametinib for melanoma was additionally prescribed with exemestane for breast cancer.

[d]Only OAT prescribed for dermato-oncological indications are shown.

[e]Includes palliative treatment with temozolomide, lenvatinib, acitretin, and adjuvant treatment with encorafenib/binimetinib.
